# Resection of rectal GIST using a novel technique: a report of two cases

**DOI:** 10.3332/ecancer.2017.760

**Published:** 2017-08-17

**Authors:** Osama Eldamshety, Islam H Metwally, Elsayed Ghoneem, Wagdi F Elkashef

**Affiliations:** 1 Surgical Oncology Unit, Oncology Center, Mansoura University (OCMU), Geehan Street, Mansoura, 35516, Egypt; 2 Hepatology and Gastroenterology Unit, Specialized Medical Hospital, Mansoura University, Gomhoria Street, Mansoura, 35516, Egypt; 3 Pathology Department, Faculty of Medicine, Mansoura University, Gomhoria Street, Mansoura, 35516, Egypt

**Keywords:** rectal tumour, GIST, TEO, imatinib, transanal

## Abstract

Rectal gastrointestinal stromal tumours (GISTs) are uncommon tumours and usually present with large sizes. We present two cases of rectal GIST. Imatinib was used in the setting of neoadjuvant and adjuvant therapy. Both tumours were resected transanally by the transanal endoscopic operation (TEO) platform. Oncosurgeons are recommended to implement sphincter-sparing surgeries for these cases.

## Introduction

Gastrointestinal stromal tumours (GISTs) are the commonest mesenchymal tumours (sarcoma) affecting the gastrointestinal tract. However, GISTs represent only less than one percent of the gut tumours [1].

Rectal GISTs are even rarer, representing 3% of all GIST cases, while most cases are detected in the stomach and small intestine (90%) [2].

The discovery of the c-Kit tyrosine kinase receptors (75–80% of cases) and the platelet-derived growth factor receptor α (PDGFRA) mutation (10–15% of cases) led to the use of Imatinib mesylate therapy. Imatinib is a tyrosine kinase inhibitor that inhibits proliferation and induces apoptosis in GIST cells [1].

However, surgery remains a cornerstone in the treatment and the only curative option for those with rectal GISTs. Several surgical options are available. In spite of this, large tumours with aggressive features have been commonly treated with abdominoperineal resection [3].

The use of transanal minimally invasive surgery (TAMIS) technique for the resection of GISTs in the rectum is documented in several studies [3,4], but to our knowledge we are the second to report local excision of rectal GIST using the transanal endoscopic operation (TEO) platform (Karl Storz, Tuttilingen, Germany).

## Case presentation

### Case one

**Figure 1.**
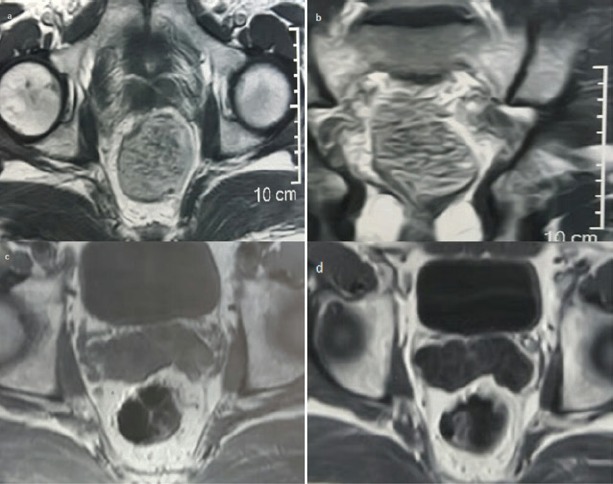
MRI of case one (a) before imatinib (axial section) (b) before imatinib (coronal section) (c) after 3 months imatinib (d) after 6 months imatinib.

**Figure 2.**
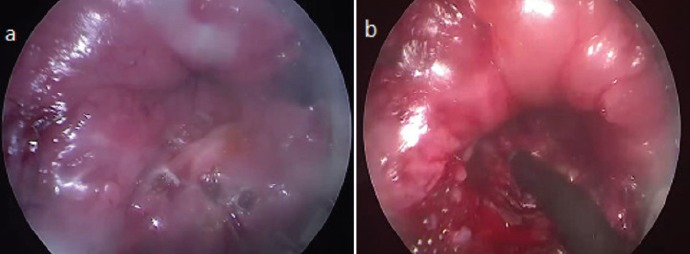
Case one (a) white thickened patch at the site of the tumour (b) Resection of the residual lesion.

A male patient aged 36 years old sought medical advice for constipation and a sense of incomplete defecation. MRI pelvis was done, which revealed a mass 8 × 3 cm at the ano-rectum. Fine-needle aspiration biopsy (FNAB) revealed a cellular spindle cell tumour, which was assigned as GIST by immunostain. Clinical oncologists started Imatinib (Gleevec®) at 400 mg daily dose for three months with good radiological response, the lesion downsized to 1.5 cm in maximum diameter (Figure 1). Another month of imatinib was implemented, followed by examination under anaesthesia, which revealed a small lesion in the right lateral wall of the anal canal 2 cm away from the anal verge. The patient was positioned in the right lateral decubitus and wide local excision using TEO platform was done, followed by primary suturing with V-Loc® suture (Covidien, MA, USA) (Figure 2). However, being a very low lesion, removal of the platform and reinforcing of the suture lines directly by absorbable interrupted sutures was needed. SURGIFLO® (Ethicon, CA, USA) was used to seal any bleeding points and a Foley’s catheter was inserted in the anal canal as a drain. Patient retained full oral and was discharged from the hospital a week later. The final pathology revealed no residual tumour, suggesting a complete pathological response to imatinib. The patient started adjuvant imatinib 400 mg daily once on full oral. A follow-up 14 months after the operations showed no evident recurrence.

### Case two

**Figure 3.**
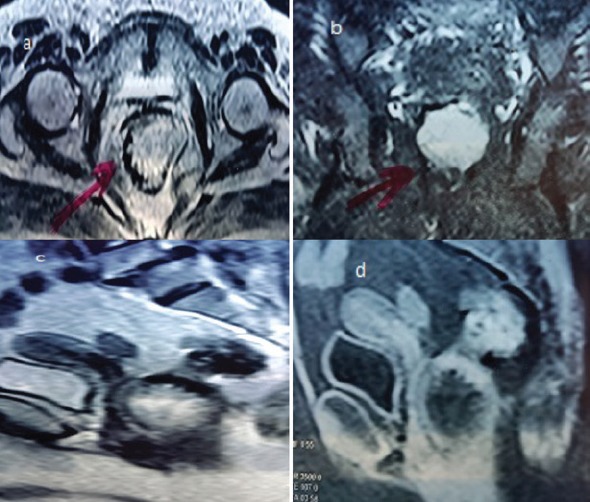
MRI of case two (a) axial section showing large tumour in the left anterolateral wall of the anal canal (b) Coronal section showing no extraluminal extension (c) T1-weighted and (d) T2-weighted sagittal views showing the tumour relation to the posterior vaginal wall.

**Figure 4.**
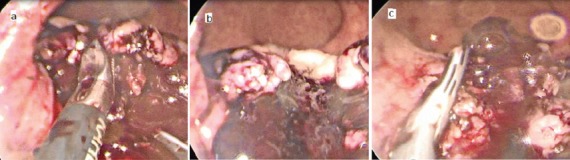
Resection of case two (a) dissection of friable tumour (b) tumour dissected completely from the left side with intact musculosa (c) cutting the remaining attachment with Harmonic scalpel.

**Figure 5.**
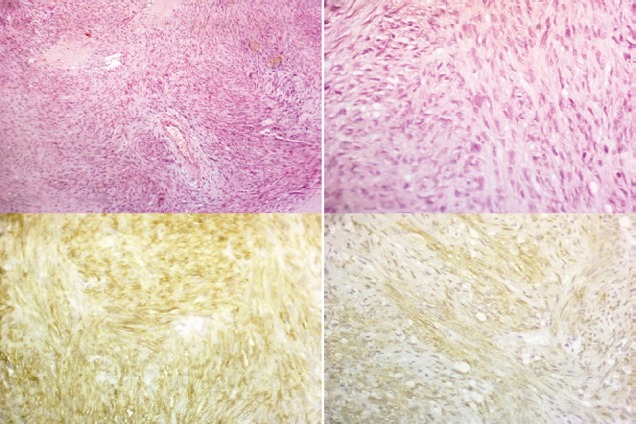
Pathology specimens of case two (a) shows interlacing bundles of spindle cells (H&E staining, 100x) (b) shows interlacing bundles of spindle cells (H&E staining, 400x) (c) shows positive membranous reaction (CD117 immunostaining, 400x) (d) shows positive membranous reaction (DOG-1 immunostaining, 400x).

A 55-year-old female patient presented to the internal medicine department with constipation and heaviness in the pelvis. Abdominal examination was free, per-rectal examination revealed a mass, 4 cm from the anal verge occupying anterior and left wall with intact overlying mucosa. Per-vaginal examination showed a palpable mass pushing the posterior vaginal wall with intact vaginal mucosa. Pelvic ultrasound showed a 5.9 × 5.6 cm mass at recto-vaginal pouch with mass effect upon the rectal lumen. Pelvic MRI reported intraluminal mass in the rectum 4.5 cm, infiltrating the left lateral rectal wall (Figure 3). Endorectal ultrasound reported a submucosal 5 cm mass, continuous with muscularis propria suggestive of an abscess or a spindle cell tumour. A colonoscopy showed ulcerating rectal lesion with an otherwise normal colon. FNAB was not diagnostic, while core needle biopsy suggested an inflammatory lesion. The multidisciplinary panel decision was transanal excision of the mass. The patient was positioned in left lateral decubitus. Complete gross excision of the mass was done by using a Harmonic scalpel® (Ethicon, CA, USA). After complete resection, further separate safety margins from the edges and depth of the cavity were sent for histopathological examination (Figure 4). Finally, suturing of defect was done by V-Loc® sutures. Pathology revealed a low-risk GIST with microscopic infiltration of left margin confirmed by immunostain (Figure 5). The patient suffered from perianal inflammation and small dehiscence in the lower part of the wound, treatment entailed injectable wide spectrum antibiotics and metronidazole. The patient improved with watchful waiting and adjuvant imatinib was prescribed for one year. The follow-up at 10 months post-operative was free.

## Discussion

Four different approaches of local resection of GISTs have been described in the literature. The transanal (Parks) and the transsacral (Kraske) approaches can achieve a complete resection in low- and mid-rectal lesions with low morbidity and mortality but usually require fragmentation of the specimen which increases the chance of local recurrence [3].

Resection of the anterior wall rectal lesions is technically difficult. Transvaginal excision was described for lesions higher on the anterior wall of the rectum, with the advantage of avoiding anal dysfunction [5]. Another method for the resection of these tumours was the perineal approach, which was implemented for GIST in the anterior wall of the rectum in some case reports [6].

More recently, transanal minimally invasive endoscopic excision of rectal GISTs has been applied as a fifth approach. This includes TEM (Richard Wolf, IL, USA) and TAMIS commonly using either GelPOINT path transanal access platform (Applied medical, CA, USA) or SILS (Covidien, MA, USA) [7]. In our cases, we used a more recent TEO platform for local excision of two rectal GISTs using the same oncologic principles of the TEM surgery (Table 1). Only one Belgian report of a similar approach was found after a thorough search [8].

In our technique, the operative field must be in a position corresponding to six o’clock of the rectoscope. The operation starts by washing the anal canal by diluted povidone-iodine and saline in order to remove residual faeces and mucous secretions. Then, gentle dilatation of the anal sphincters for a few minutes to allow insertion of the TEO platform without damage to the internal anal sphincter, followed by CO_2_ insufflation to 14 mmHg. Subsequently, we mark the excision margins by a monopolar diathermy 1 cm around the visible border of the tumour. Excision of the lesion is done by an advanced bipolar or an ultrasonic shear. Finally, we close the defect with sutures. We think the TEO platform is both feasible (comparable to TAMIS) and relatively cheap, because it is reusable (comparable to TEM). The main advantage of this technique over Kraske or Park’s technique is the delivery of an intact specimen.

Rectal and anal GISTs are rare and thought to be of worse prognosis. Limited evidence from case reports and small case series suggests that neoadjuvant imatinib therapy can successfully downsize tumours and aids organ-preserving surgery in patients with high-risk rectal GISTs [9]. One of our cases was unique in attaining complete pathological response to imatinib neoadjuvant therapy, while the other one could not be diagnosed pre-operatively and so upfront surgery was implemented.

## Conclusion

Sphincter-sparing procedures for rectal GISTs using minimally invasive techniques should be the treatment of choice whenever possible. The TEO platform is an adequate method for achieving this goal. Imatinib as a neoadjuvant therapy for rectal GIST is a promising option, which increases the chance of tumour downstaging and thus anal sphincter preservation.

## Conflict of interest

The authors declare no conflict of interest.

## Figures and Tables

**Table 1.** A comparison between different market available platforms for transanal endoscopic surgery (TES).

	TEM	TAMIS	TEO
Date invented	1983	2009	2003
Type	Rigid	Flexible	Rigid, now B-port allows some flexibility
Need for assistance	No	Need assistant	No
Durability	Reusable	One use	reusable
Vision	Binocular but now with 3D stereoscope	Uniocular	Uniocular
Lens	Special stereoscope	Conventional laparoscopic telescope	Special telescope
Size	Rectoscopes of 12, 13.7, 20 cm length	Many platforms, commonest SILS and GelPOINT path	Available in three sizes 7.5, 15, 20 cm rectoscopes
Angle of vision	45 degrees	Straight	70 degrees
Fixation	To table	Gelpoint is fixed by sutures. However SILS is not stable.	To table
Cost	Highest	Least but being non-reusable makes it the highest in long run.	Less than TEM
Pressure fluctuation	Least due to special insufflations system.	High but recently Airseal ®(ConMed, NY, USA) is used to stabilize the pressure.	High but recently B-port with high flow adaptor minimizes the fluctuation.
Instrumentation	Special curved tipped	Standard laparoscopic	Special curved tipped
